# Susceptibility of Poultry to Pandemic (H1N1) 2009 Virus

**DOI:** 10.3201/eid1512.091060

**Published:** 2009-12

**Authors:** David E. Swayne, Mary Pantin-Jackwood, Darrell Kapczynski, Erica Spackman, David L. Suarez

**Affiliations:** US Department of Agriculture, Athens, Georgia, USA

**Keywords:** Influenza, pandemic (H1N1) 2009 virus, viruses, expedited, poultry, Japan, quail, letter

**To the Editor:** During April 2009, cases of acute respiratory disease in humans caused by influenza A pandemic (H1N1) 2009 virus in Mexico were reported (*1*). By August 21, 2009, a total of >182,166 human cases, including 1,799 deaths, had been reported from 177 countries (www.who.int/csr/don/2009_08_21/en/index.html).

The origin of the new virus appears to be a reassortant event of a virus from swine in North America that contained the classic swine, human, and avian influenza genes and a virus of unknown origin that contributed neuraminidase and matrix genes of swine in Europe. On May 2, 2009, the first nonhuman infections were detected in a swine operation in Canada (www.who.int/csr/don/2009_06_24/en/index.html).

Historically, human seasonal influenza A viruses have not been reported to infect poultry, but clinical cases of respiratory disease or reduction in egg production have been reported for domestic turkeys after infection with subtypes H1N1, H1N2, and H3N2 swine influenza viruses and for multiple poultry species with subtype H1N1 avian influenza virus (*2**–**4*). The presence of avian and swine influenza virus genes in pandemic (H1N1) 2009 virus increases the potential for infection in poultry after exposure to infected humans or swine.

To determine infectivity potential, 3-week-old chickens (*Gallus domesticus*) (n = 11), 2-week-old domestic ducks (*Anas platyrhynchos*) (n = 11), 73-week-old reproductively active turkey hens (*Meleagris gallopavo*) (n = 9), 3-week-old turkey poults (n = 11), and 5-week-old Japanese quail (*Coturnix japonica*) (n = 11) were intranasally inoculated with 10^6^ mean chicken embryo infectious doses of A/Mexico/4108/2009(H1N1). Five uninfected chickens, ducks, turkey poults, and quail, and 3 uninfected turkey hens were contact exposed to intranasally inoculated birds to assess transmission potential. Cloacal and oropharyngeal swabs were taken on 2, 4, 7, and 10 days postinoculation (DPI) from all birds, and internal tissues were taken from 2 birds on 2, 4 and 7 DPI for virus detection by quantitative real-time reverse transcription–PCR (qRRT-PCR) assay specific for the influenza virus matrix gene (*5*).

To improve sensitivity because of several primer mismatches, we updated the reverse primer to 3′-cagagactggaaagtgtctttgca-5′. Virus isolation in embryonating chicken eggs was used on a subset of samples to verify qRRT-PCR results at 4 DPI. Serum samples were collected on 15 DPI for antibody testing by hemagglutination inhibition. Shams were intranasally inoculated with culture media and sampled on 4 and/or 7 DPI. We inoculated ten 4 week-old chickens intravenously to determine pathotype by using the intravenous pathogenicity index (IVPI). All animal studies were conducted under BioSafety Level 3 enhanced conditions with approval by Institutional Animal Care and Use and BioSafety committees.

During the 15-day observation period, clinical signs did not develop in any of the birds; none of the birds died. An IVPI of 0.00 indicated the virus was not of high pathogenicity for chickens. No virus was detected by qRRT-PCR or isolated in chicken eggs from swabs or tissues from chickens, turkeys, or ducks. All chickens and turkeys were negative for antibodies to the virus on 15 DPI, but 1 intranasally inoculated duck had a hemagglutination inhibition (HI) antibody titer of 16. Virus was detected in oropharyngeal swabs at 2 and 4 DPI from intranasally (IN)–inoculated quail (Table), and these quail had antibodies against influenza A at 15 DPI. The intranasally inoculated quail had heterophilic-to-lymphocytic rhinitis, and influenza virus was visualized by immunohistochemical analysis of epithelium and macrophages within the mucosa of the nasal cavity; neither lesions nor antigen were identified in other respiratory and nonrespiratory tissues. Virus was not isolated from contact-exposed quail (Table), and they lacked antibodies on 15 DPI.

**Table:** Results of testing for influenza A pandemic (H1N1) 2009 virus in oropharyngeal swabs of experimental quail

Group	Sampling day (days postinoculation) for oropharyngeal swab*			
	2	4	7	10
Intranasally inoculated	2/5 (10^0.9^)	5/5 (10^2.8^)	0/5	0/5
Contact	0/5	0/5	0/5	0/5

*Number virus positive/total sampled (average titer of positive samples, mean chicken embryo infectious doses). Test results for all cloacal swabs were negative.

Infection with swine influenza viruses in turkeys has been frequently reported, and experimental intranasal inoculation studies using 5 such viruses have produced infection and disease with associated contact transmission to uninfected turkeys (*3**,**4**,**6*). However, infection of chickens by swine influenza viruses has been rare in the field, and experimental studies have shown limited respiratory replication after intranasal inoculation but no transmission (*3**,**6**–**8*). Experimental inoculation of ducks failed to produce infection or transmission (*8*).

Recently, subtype H3N2 swine influenza A virus infection with respiratory disease in Japanese quail has been reported in Canada, and such infections have been experimentally reproduced by intranasal inoculation (*9**,**10*). However, in our studies, pandemic (H1N1) 2009 virus was biologically distinct from swine influenza viruses, failing to produce infection in experimentally inoculated turkey hens or chickens, and only 1 serologically positive IN-inoculated domestic duck. In addition, Japanese quail were infected by high dose IN exposure, but replication and shedding was limited to the respiratory tract, and the virus did not transmit to quail by contact, suggesting low potential of poultry involvement as an amplification host for current pandemic (H1N1) 2009 virus. Pandemic (H1N1) 2009 virus is unlikely to produce sustained outbreaks in poultry unless the virus mutates or reassorts with existing avian influenza viruses. Since the submission of this report, the virus has been detected in 2 turkey flocks in Chile (www.oie.int/wahis,/public.php?page=single_report&pop=1&reportid=8404). Currently, only limited data are available, and it is unknown if pandemic (H1N1) 2009 has changed and acquired the ability to infect and transmit in turkeys or if the 2 cases are isolated events without epidemic potential in turkeys.
